# Rapid restructuring of surface-water bacterial communities across the spring-to-early-summer transition in a water-conservancy reservoir

**DOI:** 10.3389/fmicb.2026.1992256

**Published:** 2026-10-08

**Authors:** Ding Li, Yijun Liu, Xiaoping Wu, Yulin Lu, Xinyu Wu, Xiyao Liu

**Affiliations:** 1 College of Marine Sciences, Ningde Normal University, Ningde, China; 2 Ningde Marine Center, Ministry of Natural Resources, Ningde, China; 3 Fujian Provincial Key Laboratory of Featured Biochemical and Chemical Materials, College of New Energy and Materials, Ningde Normal University, Ningde, China

**Keywords:** 16S rRNA gene sequencing, bacterioplankton, community turnover, *Cyanobium*, temporal succession

## Abstract

Reservoirs are engineered freshwater ecosystems in which seasonal environmental variability interacts with the physical and physicochemical conditions created by water-conservancy infrastructure. However, bacterial succession during relatively short seasonal transitions remains insufficiently resolved in many subtropical reservoirs. Here, we investigated surface-water bacterial communities in Guanchang Reservoir, southeastern China, across eight sampling campaigns from 14 April to 23 June 2026. Thirty-two samples were collected from four fixed stations spanning the reservoir, including the reservoir-head area, an intermediate station, the water-intake zone, and the dam-front area. Bacterial communities were characterized using 16S rRNA gene amplicon sequencing and examined together with water temperature, pH, dissolved oxygen, ammonium nitrogen, total phosphorus, and total nitrogen. Bacterial richness and Shannon diversity differed significantly among sampling dates. Community composition exhibited pronounced temporal succession, with non-metric multidimensional scaling showing clear differentiation among sampling periods (stress = 0.098, R = 0.7654, *P* = 0.001). *Pseudomonadota*, *Actinomycetota*, *Bacteroidota*, *Cyanobacteriota*, *Bacillota*, *Planctomycetota*, and *Verrucomicrobiota* dominated the assemblages, but substantial turnover occurred at finer taxonomic levels. A particularly conspicuous transition occurred in early June, when *Cyanobium* PCC-6307 increased simultaneously across all four stations, whereas several taxa characteristic of earlier communities, including *Clostridium*, *Turicibacter*, *Terrisporobacter*, *Polynucleobacter*, and the CL500-29 marine group, declined. Canonical correspondence analysis linked bacterial turnover to changing environmental gradients, while genus-level correlations showed contrasting associations of spring- and early-summer-associated taxa with temperature, dissolved oxygen, and nutrient concentrations. These findings demonstrate rapid and coordinated bacterial succession during the spring-to-early-summer transition in a regulated reservoir and highlight the ecological importance of resolving transient microbial states within water-conservancy ecosystems.

## 1 Introduction

Reservoirs represent a distinctive category of freshwater ecosystems created and maintained by dams, water storage, withdrawal, flow regulation, and other hydraulic operations. Compared with free-flowing rivers, reservoir formation generally reduces current velocity, increases water residence time, alters longitudinal connectivity, enhances sediment and nutrient retention, and modifies thermal and oxygen structures. These changes influence both microbial dispersal and local environmental selection and can therefore reshape aquatic microbial communities and their ecological processes (Lindström and Bergström, 2004; Lindström et al., 2005; Niño-García et al., 2016). Studies of dam-regulated rivers and cascade reservoirs have further demonstrated that hydraulic engineering can alter microbial community composition, interaction structure, and potential ecosystem functions through changes in hydrological and physicochemical conditions (Chen et al., 2018; Shi et al., 2020; Li et al., 2022; Jiang et al., 2023; Wang et al., 2023).

Bacterioplankton are central components of freshwater food webs and participate extensively in organic-matter transformation, nutrient regeneration, and elemental cycling. Although freshwater bacterial communities commonly contain recurrent phylogenetic lineages, their relative abundances and fine-scale composition can change considerably through time (Newton et al., 2011). Temporal variation is therefore a fundamental dimension of freshwater microbial diversity. Long-term investigations have demonstrated repeatable bacterial phenology and strong seasonal turnover in lakes, with temperature, nutrient availability, water-column mixing, and biological interactions frequently associated with community succession (Shade et al., 2007; Kara et al., 2013; Yan et al., 2017).

Seasonal bacterial succession is also widely documented in reservoirs. Studies of subtropical reservoirs in China have shown pronounced temporal variation in bacterioplankton community composition even among systems with similar trophic conditions (Li et al., 2016). Seasonal variability can affect abundant, rare, and conditionally rare bacterial fractions simultaneously, indicating that temporal environmental change restructures multiple components of reservoir microbiomes (Nyirabuhoro et al., 2020). In Danjiangkou Reservoir, seasonal shifts in bacterioplankton composition were closely associated with environmental variation, including nitrogen and phosphorus availability (Chen et al., 2022). More recent investigations of plateau and drinking-water reservoirs have likewise identified strong temporal community restructuring associated with temperature and water-quality variation (Wu et al., 2024; Yang et al., 2024).

Most seasonal studies, however, compare broadly defined spring, summer, autumn, and winter communities. Such designs may overlook microbial transitions occurring over much shorter intervals. Freshwater bacterial communities can reorganize substantially within weeks, and short-term temporal variation can contribute strongly to beta diversity (Jones et al., 2012; Paruch et al., 2020). Seasonal transition periods are particularly relevant because water temperature, nutrient availability, light, water-column stability, and phytoplankton composition may change simultaneously, potentially generating transient microbial states that are not captured by low-frequency sampling.

*Cyanobacteria* provide an especially important link between seasonal microbial succession and reservoir water-quality dynamics. Freshwater *Cyanobacteria* communities respond to interacting effects of temperature, nutrient availability, light, stratification, mixing, and hydrological residence time (Paerl and Otten, 2013; Paerl et al., 2016; Visser et al., 2016; Huisman et al., 2018). Picocyanobacteria such as *Cyanobium* are widespread components of freshwater plankton and can exhibit pronounced seasonal and vertical niche differentiation (Callieri, 2010; Nwosu et al., 2021; Carratalà et al., 2023). Temporal increases in *Cyanobium* may therefore represent an informative component of cyanobacterial community restructuring, although relative abundance obtained from 16S rRNA gene sequencing cannot by itself demonstrate bloom biomass or toxin production.

Guanchang Reservoir is located in Ningde, Fujian, in southeastern China, and is the drinking water source reservoir for Ningde City. The reservoir includes a water-intake zone where a bottom-aeration system has been deployed for water-quality management and bloom prevention. Rather than treating this engineering intervention as an experimental manipulation, the present study focused on bacterial succession within the broader regulated reservoir ecosystem. Four fixed surface-water stations were repeatedly sampled on eight dates between mid-April and late June 2026.

The present study was designed to resolve bacterial community dynamics at a finer temporal scale than conventional comparisons among broadly separated seasons. By repeatedly sampling four fixed stations on eight occasions within a single spring-to-early-summer transition, we aimed to determine whether short-lived intermediate community states could be detected during this relatively narrow temporal window. Specifically, we investigated: (1) whether bacterial richness, diversity, and community structure changed systematically during the transition; (2) which dominant bacterial taxa and ASVs contributed most strongly to temporal turnover; and (3) how these compositional changes were associated with the measured physicochemical environment. We expected that the spring-to-early-summer transition would be accompanied by pronounced bacterial community reorganization and that concurrent changes in thermal and nutrient conditions would be associated with shifts among dominant freshwater bacterial populations.

## 2 Materials and methods

### 2.1 Study area and sampling design

The study was conducted in Guanchang Reservoir, located in Badu Town, Jiaocheng District, Ningde, Fujian Province, southeastern China. Four fixed surface-water sampling stations were selected to represent different positions within the reservoir: KT, located in the reservoir-head area; KX, located at the middle of the reservoir’s diversion point; QSK, located in the water-intake zone; and BQ, located in the dam-front area. A bottom-aeration system was deployed near the QSK water-intake station as a local engineering measure for water-quality improvement and bloom prevention. Because the sampling design did not include pre-intervention observations or replicated aerated and non-aerated experimental units, QSK was treated as a fixed spatial station rather than as an experimental treatment.

Surface-water samples were collected simultaneously from all four stations on 14 April, 24 April, 30 April, 7 May, 14 May, 5 June, 11 June, and 23 June 2026, resulting in a total of 32 microbial samples (Figure 1). Water was collected from the reservoir surface using sterile water sampling bottle. Approximately 1 L of water was filtered through a sterile 0.22 μm membrane filter, and the filters were stored at −80 °C until DNA extraction.

**FIGURE 1:**
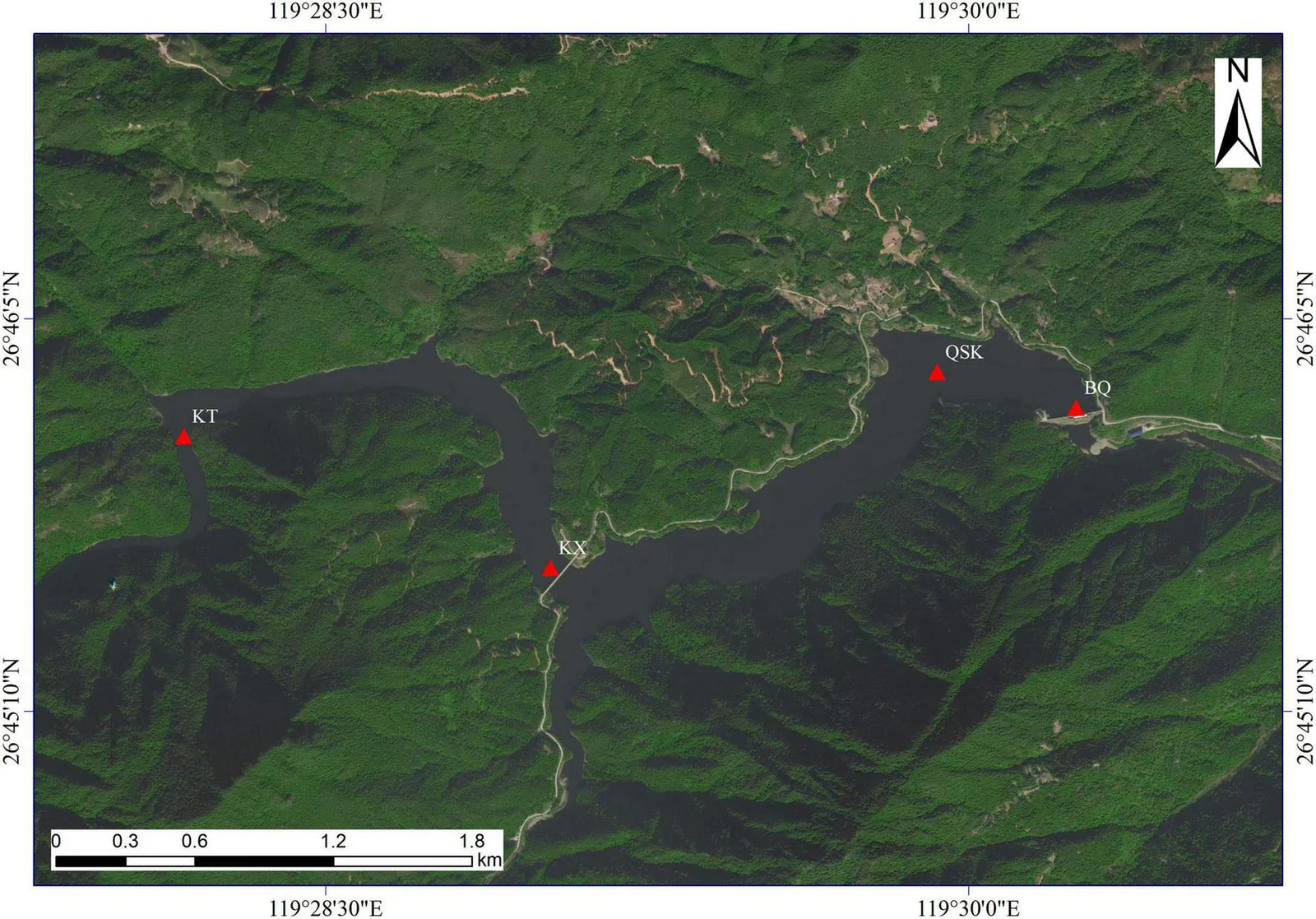
Location of Guanchang Reservoir and the four surface-water sampling stations. Guanchang Reservoir is located in Badu Town, Jiaocheng District, Ningde, Fujian Province, southeastern China. Four fixed sampling stations were included: KT, located in the reservoir-head area; KX, located approximately 200 m from the reservoir head; QSK, located in the water-intake zone; and BQ, located in the dam-front area. Surface-water samples were collected from all four stations on eight sampling dates between 14 April and 23 June 2026.

### 2.2 Physicochemical measurements

Water temperature, pH, and dissolved oxygen (DO) were measured *in situ* using a DZB-718 portable multiparameter water-quality meter (Shanghai INESA Scientific Instrument Co., Ltd., Shanghai, China). Ammonium nitrogen (NH_4_^+^-N) was determined using the Nessler’s reagent spectrophotometric method according to HJ 535-2009. Total phosphorus (TP) was determined using the ammonium molybdate spectrophotometric method following GB 11893-89 after potassium persulfate digestion, whereas total nitrogen (TN) was measured using the alkaline potassium persulfate digestion UV spectrophotometric method according to HJ 636-2012.

### 2.3 DNA extraction, PCR amplification, and sequencing

Surface-water samples were collected simultaneously from all four stations on 14 April, 24 April, 30 April, 7 May, 14 May, 5 June, 11 June, and 23 June 2026, resulting in 32 microbial samples (Figure 1). Water samples were collected from the surface layer using sterile water-sampling bottles between 17:00 and 17:30 during each sampling campaign. After collection, the samples were immediately placed on ice and transported to the laboratory for filtration. Microbial genomic DNA was extracted directly from the 0.22 μm membrane filters obtained from each 1 L surface-water sample using the DNeasy PowerSoil Pro Kit, following the manufacturer’s instructions (QIAGEN, GmbH, Hilden, Germany). The concentration of the extracted DNA was measured using a Qubit 3.0 Fluorometer (Life Technologies, Thermo Fisher Scientific, Waltham, MA, USA) and the Equalbit 1× dsDNA HS Assay Kit (Vazyme, Nanjing, China). DNA quality and concentration were further evaluated using 1.0% agarose gel electrophoresis and a NanoDrop ND-2000 spectrophotometer (Thermo Scientific, Waltham, MA, USA).

The primer set 515FmodF (5′-GTGYCAGCMGCCGCG GTAA-3′) and 806R (5′-GGACTACHVGGGTWTCTAAT-3′) was used to amplify the V4 region of prokaryotic 16S rRNA genes, covering bacterial and archaeal lineages where amplified. PCR was conducted in a 20 μL reaction volume, including 10 ng of DNA, 0.8 μL of each primer (10 μM), 4 μL 5× FastPfu buffer (Vazyme, Nanjing, China), 0.4 μL FastPfu polymerase (Vazyme, Nanjing, China), 0.2 μL BSA (Vazyme, Nanjing, China), and 250 μM dNTPs (Vazyme, Nanjing, China). PCR cycling conditions were as follows: initial denaturation at 95 °C for 3 min, followed by 29 cycles of denaturation at 95 °C for 30 s, annealing at 55 °C for 30 s, extension at 72 °C for 45 s, and a final extension at 72 °C for 10 min. Purified amplicons were combined in equimolar proportions and sequenced using an Illumina MiSeq PE300 platform, following the standard protocols provided by Majorbio Bio-Pharm Technology Co., Ltd., (Shanghai, China).

### 2.4 Sequence processing and taxonomic assignment

Raw sequencing reads were demultiplexed according to sample-specific barcodes and processed using QIIME 2. Raw paired-end sequences were processed using the DADA2 plugin in QIIME 2 with the default parameters. No bases were trimmed from the 5′ ends of the forward or reverse reads (trim−left−f = 0 and trim−left−r = 0), and no fixed-length truncation was applied (trunc−len−f = 0 and trunc−len−r = 0). Reads with more than two expected errors in either the forward or reverse direction were discarded (max−ee−f = 2.0 and max−ee−r = 2.0). Reads were truncated at the first position with a quality score ≤ 2 (trunc−q = 2). Paired-end reads were merged using a minimum overlap of 12 bp, with no mismatches allowed in the overlapping region. Samples were denoised independently, and chimeric sequences were identified and removed using the consensus method. The resulting amplicon sequence variants were used for downstream analyses. Taxonomic assignment of representative ASV sequences was performed using the classify-sklearn method implemented in the q2-feature-classifier plugin of QIIME 2. A Naive Bayes classifier was used to assign taxonomy against the SILVA 16S rRNA gene reference database (release 138.2), with a confidence threshold of 0.70. Following taxonomic assignment, ASVs classified as chloroplasts or mitochondria were removed before downstream analyses. No archaeal ASVs were detected in the dataset; therefore, the sequences retained for subsequent community analyses represented bacterial ASVs. Community ecological analyses, including diversity analysis, ordination analysis, PERMANOVA, and multivariate-dispersion analysis, were conducted using the vegan package (version 2.6-8).

A total of 2,184,240 raw reads were generated from the 32 samples. After initial quality filtering, 2,030,170 high-quality reads were retained, corresponding to 92.95% of the raw reads. Following DADA2 denoising and sequence processing, 1,734,684 reads remained for downstream analyses, representing 79.42% of the original sequencing reads. The final sequencing depth ranged from 40,255 to 74,945 reads per sample, and the number of ASVs detected in individual samples ranged from 347 to 755, indicating adequate sequencing coverage across the dataset.

### 2.5 Diversity and community composition analyses

Prior to downstream community analyses, the ASV abundance table was rarefied to an even sequencing depth of 35,774 reads per sample to minimize potential biases associated with unequal sequencing depth. Bacterial alpha diversity was evaluated at the ASV level using Sobs richness, Shannon diversity, and Good’s coverage calculated from the rarefied ASV table. Differences in alpha-diversity indices among the eight sampling dates were assessed using Kruskal–Wallis H tests. Shared and sampling-date-specific ASVs were visualized using Venn diagrams based on the rarefied dataset. Taxonomic composition was summarized at the phylum, class, family, and genus levels using relative abundances calculated from the rarefied ASV table, without prior removal of low-abundance taxa. Beta-diversity patterns were evaluated using non-metric multidimensional scaling (NMDS) based on Bray–Curtis dissimilarities calculated from the rarefied ASV abundance table. Differences in overall community composition among sampling-date groups were assessed using ANOSIM. Differences in the relative abundances of bacterial taxa among sampling dates were evaluated using Kruskal–Wallis H tests.

### 2.6 Differential taxa and environmental association analyses

The 50 most abundant ASVs were visualized using hierarchical clustering and heatmap analysis to characterize temporal abundance patterns. Temporal differences in individual ASVs, bacterial families, and genera were evaluated using Kruskal–Wallis H tests. Canonical correspondence analysis (CCA) was performed to examine relationships between bacterial community composition and water temperature, pH, DO, NH_4_^+^-N, TP, and TN. Spearman rank correlations were further calculated between dominant bacterial genera and the measured environmental variables.

### 2.7 Nucleotide accession numbers

The raw sequence data are available in the Genome Sequence Archive (Genomics, Proteomics & Bioinformatics 2021) at the National Genomics Data Center (Nucleic Acids Res 2022). Data availability for peer review: CRA049302.^1^

Editors and reviewers can make data availability inquiries within 3 months.

## 3 Results

### 3.1 Environmental variation during the spring-to-early-summer transition

The physicochemical environment of Guanchang Reservoir changed substantially during the sampling period from mid-April to late June 2026. Water temperature exhibited a pronounced but non-monotonic temporal pattern. Mean surface-water temperature was approximately 23.7 °C on 14 April and subsequently decreased to approximately 18.3 °C on both 24 and 30 April. Temperature then increased to approximately 20.2 °C on 7 May and continued to rise during June, reaching approximately 23.5 °C on 5 June and 25.8 °C on 11 June. By 23 June, mean surface-water temperature had increased sharply to approximately 33.3 °C, representing the warmest conditions recorded during the study period.

Compared with temperature, dissolved oxygen exhibited considerably less temporal variation across the sampling period. Mean DO values were approximately 8.08 and 8.03 mg/L on 14 and 24 April, respectively, and remained around 8.10 mg/L on 7 May. DO increased moderately in June, reaching approximately 8.40 mg/L on 5 June and 8.75 mg/L on 11 June, before decreasing slightly to approximately 8.40 mg/L on 23 June. Surface-water pH was generally close to neutral and varied within a relatively narrow range throughout the sampling period. Mean pH was approximately 7.03 on both April sampling dates, increased slightly to approximately 7.15 on 7 May and 5 June, temporarily decreased to approximately 6.68 on 11 June, and subsequently returned to approximately 7.15 on 23 June.

Nutrient concentrations displayed a clearer overall temporal decline. Mean total phosphorus decreased from approximately 0.235 mg/L on 14 April to 0.153 mg/L on 24 April and 0.133 mg/L on 30 April. After minor fluctuations during May, TP declined further to approximately 0.095 mg/L on 5 June, 0.088 mg/L on 11 June, and 0.070 mg/L on 23 June. Total nitrogen followed a broadly similar trajectory, decreasing from approximately 2.15 mg/L on 14 April to 1.40 mg/L on 24 April and 1.35 mg/L on 30 April. TN remained around 1.3–1.4 mg/L during May before decreasing to approximately 1.10 mg/L on 5 June and 0.96 mg/L by 23 June.

NH_4_^+^-N also decreased overall, although its temporal pattern was less monotonic than those of TN and TP. Mean NH_4_^+^-N declined from approximately 0.265 mg/L on 14 April to 0.223 mg/L on 24 April and 0.171 mg/L on 30 April. Concentrations decreased further to approximately 0.167 and 0.132 mg/L on 7 and 14 May, respectively, followed by a modest increase to approximately 0.188 mg/L on 5 June. Values then decreased again to approximately 0.147 mg/L during the final two sampling campaigns.

Overall, the environmental transition from spring to early summer was characterized by an initial cooling phase in late April followed by pronounced warming during June, while TN and TP showed progressive declines across the study period. Thus, the bacterial community experienced a changing environmental background in which warmer early-summer conditions coincided with substantially lower bulk nitrogen and phosphorus concentrations than those observed during the initial April sampling.

### 3.2 Temporal variation in bacterial richness and diversity

Bacterial alpha diversity showed significant temporal variation across the eight sampling dates. The observed richness represented by the Sobs index differed significantly among temporal groups according to the Kruskal–Wallis H test (*P* = 0.00764). Richness was relatively high during the first two sampling campaigns in April, declined during late April and May, and subsequently increased again toward late June. The temporal pattern therefore did not represent a simple progressive gain or loss of bacterial taxa but instead showed a decline followed by recovery during the spring-to-early-summer transition.

Shannon diversity also varied significantly among sampling dates (*P* = 0.01497). Relatively high Shannon values were observed during late April, whereas diversity generally decreased during portions of May and early June. The early-June samples, particularly those collected around 5 June, showed comparatively low diversity, followed by a marked increase by 23 June. The difference between Sobs and Shannon trajectories indicates that temporal variation involved not only changes in the number of detectable ASVs but also changes in community evenness and dominance.

Good’s coverage remained consistently close to 1 across all sampling dates, indicating that sequencing depth captured the majority of detectable bacterial diversity. Although the Kruskal–Wallis test indicated a statistically significant difference in coverage among dates (*P* = 0.03605), absolute differences were very small compared with those observed for richness and Shannon diversity. Thus, temporal differences in community diversity were unlikely to have resulted from substantial differences in sequencing coverage (Figure 2).

**FIGURE 2:**
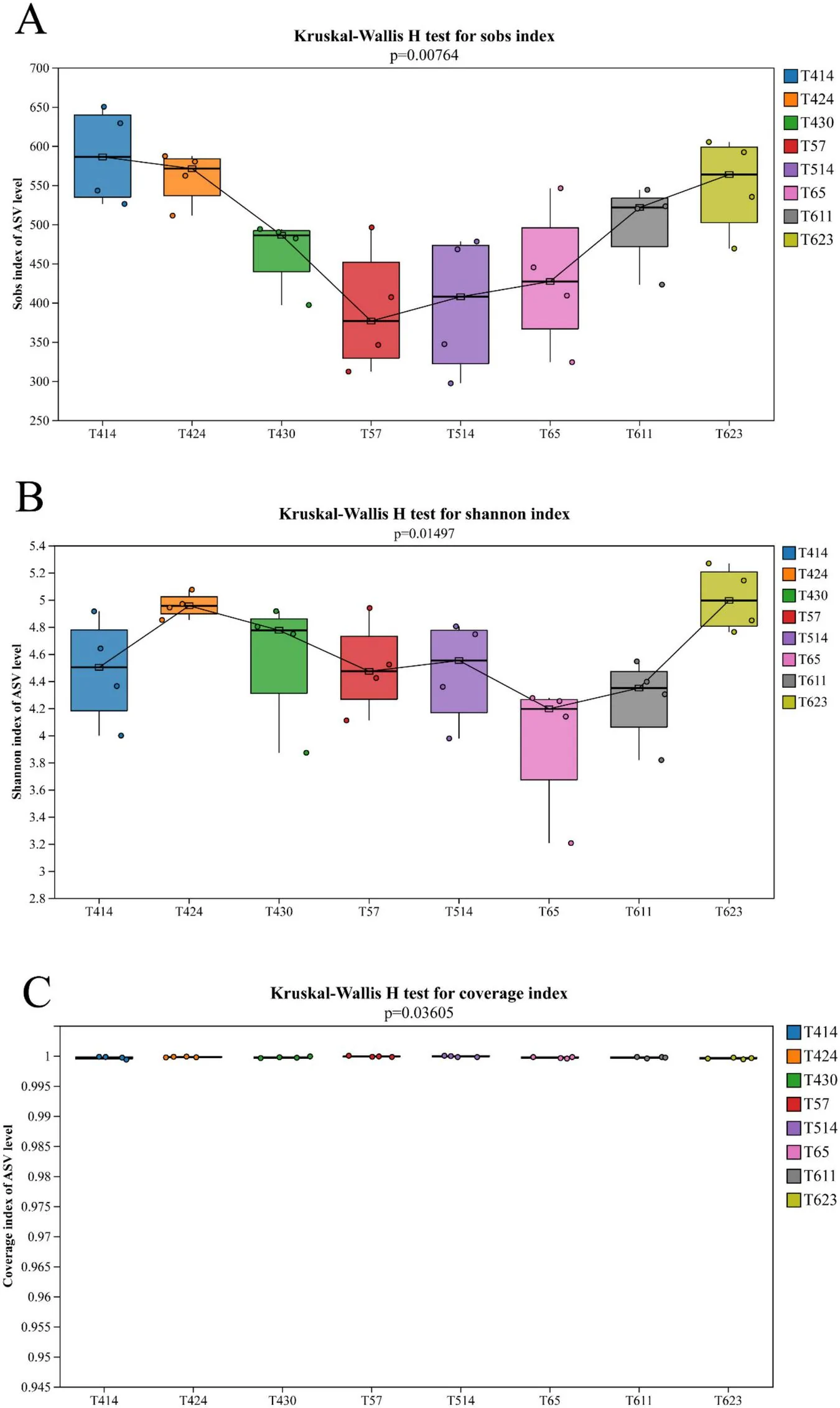
Temporal variation in bacterial alpha diversity in surface waters of Guanchang Reservoir. **(A)** Sobs richness, **(B)** Shannon diversity, and **(C)** Good’s coverage at the ASV level across the eight sampling dates. T414, T424, T430, T57, T514, T65, T611, and T623 represent samples collected on 14 April, 24 April, 30 April, 7 May, 14 May, 5 June, 11 June, and 23 June 2026, respectively. Each point represents one sampling station. Differences among sampling dates were evaluated using the Kruskal–Wallis H test. Significant temporal differences were detected for Sobs (*P* = 0.00764), Shannon diversity (*P* = 0.01497), and Good’s coverage (*P* = 0.03605).

The Venn analysis further revealed substantial temporal variation in ASV detection. At the ASV level, 195 ASVs were detected during all eight sampling periods, indicating the persistence of a shared detectable bacterial component throughout the study. At the same time, substantial numbers of ASVs were detected only during individual sampling dates. Specifically, 495 ASVs were detected only on 14 April, followed by 263 on 24 April, 136 on 30 April, 96 on 7 May, 137 on 14 May, 122 on 5 June, 148 on 11 June, and 270 on 23 June (Supplementary Figure 1). The temporal pattern in the number of sampling-date-specific detections broadly corresponded with the Sobs richness pattern. Because detection of low-abundance ASVs can depend on sequencing depth and stochastic sampling, these date-specific ASVs are interpreted as taxa detected only at particular sampling dates rather than as direct evidence of ecological appearance, disappearance, or recruitment.

### 3.3 Pronounced temporal succession of bacterial community structure

Temporal differences were even more evident at the level of overall community composition. NMDS analysis at the ASV level produced a stress value of 0.098, indicating that the two-dimensional ordination provided a reliable representation of among-sample community dissimilarity. Samples grouped according to sampling date showed strong differentiation, with an associated statistic of R = 0.7654 and *P* = 0.001.

The ordination revealed a clear directional temporal pattern. Samples collected during April were located predominantly toward the negative region of NMDS1, whereas May communities occupied more intermediate positions. This gradual movement was followed by a pronounced shift during early June, when samples collected on 5 and 11 June were positioned mainly toward positive NMDS1 values. Communities sampled on 23 June were again separated from the early- and mid-June assemblages, indicating that bacterial composition continued to reorganize after the initial early-summer shift rather than remaining in a stable June state (Figure 3). The temporal displacement observed in NMDS space was therefore characterized by several successive community states. Early- and late-April samples represented one end of the observed compositional gradient, May samples formed intermediate assemblages, and early-June communities showed the greatest shift away from spring conditions. The final 23 June assemblages occupied another distinct position, demonstrating further reorganization within early summer.

**FIGURE 3:**
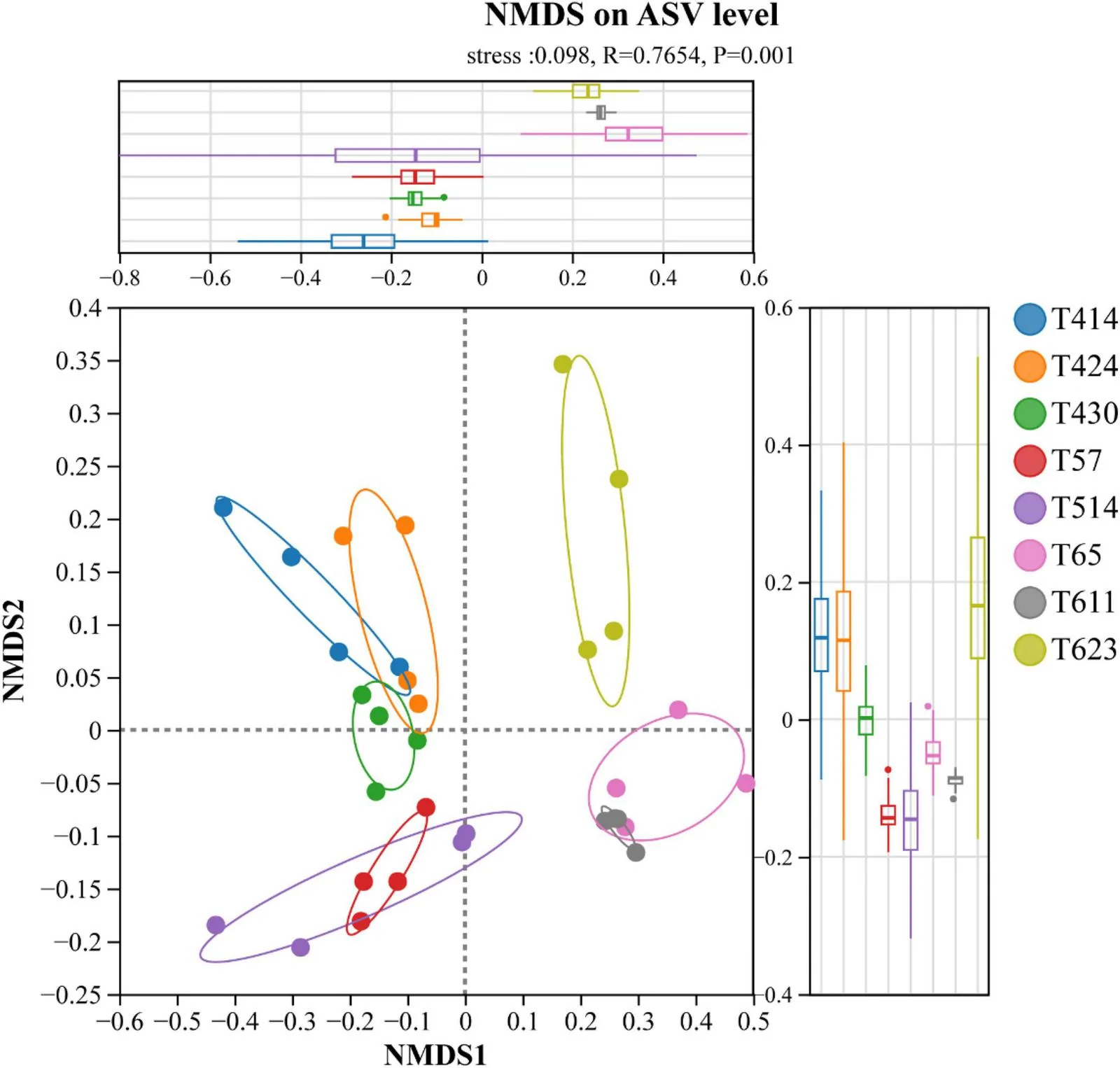
Temporal succession of surface-water bacterial communities in Guanchang Reservoir based on NMDS. NMDS ordination was performed at the ASV level using Bray–Curtis dissimilarities for the 32 surface-water samples collected across eight sampling dates. Colors represent different sampling dates, and each point represents an individual station-level sample. Ellipses summarize the distribution of samples within each sampling-date group in the ordination space. Marginal boxplots show the distributions of NMDS1 and NMDS2 scores among sampling-date groups. Differences in community composition among sampling dates were evaluated using ANOSIM (R = 0.7654, *P* = 0.001).

Importantly, the NMDS pattern was consistent with the changes observed in alpha diversity. The community state around early June, which was clearly separated in the ordination, also corresponded to reduced Shannon diversity and a marked increase in the dominance of particular bacterial taxa. By late June, increased Shannon diversity coincided with another shift in ordination space. Together, these results indicate that temporal succession involved simultaneous changes in community membership, relative abundance structure, and overall compositional dissimilarity.

### 3.4 Temporal turnover in bacterial taxonomic composition

Despite pronounced temporal variation, the surface-water bacterial assemblage retained a broadly consistent higher-level freshwater taxonomic structure. *Pseudomonadota*, *Actinomycetota*, *Bacteroidota*, *Cyanobacteriota*, *Bacillota*, *Planctomycetota*, and *Verrucomicrobiota* constituted the principal bacterial phyla throughout the study period. Other groups, including *Armatimonadota*, *Deinococcota*, *Bdellovibrionota*, *Chloroflexota*, *Myxococcota*, and *Acidobacteriota*, generally occurred at lower relative abundances. *Pseudomonadota* contributed substantially to most samples and remained one of the dominant phyla throughout the time series. *Actinomycetota* was similarly persistent across dates, although its relative contribution varied among sampling periods. *Bacillota* was conspicuous in several April samples but decreased substantially toward May and June. In contrast, *Cyanobacteriota* showed one of the strongest temporal changes. Its relative abundance was comparatively low during most April and May samples but increased markedly during early June, particularly on 5 and 11 June. This increase occurred at multiple sampling stations. By 23 June, however, *Cyanobacteriota* had declined again, while *Pseudomonadota*, *Actinomycetota*, *Bacteroidota*, and other bacterial groups regained larger proportions of the community (Figure 4).

**FIGURE 4:**
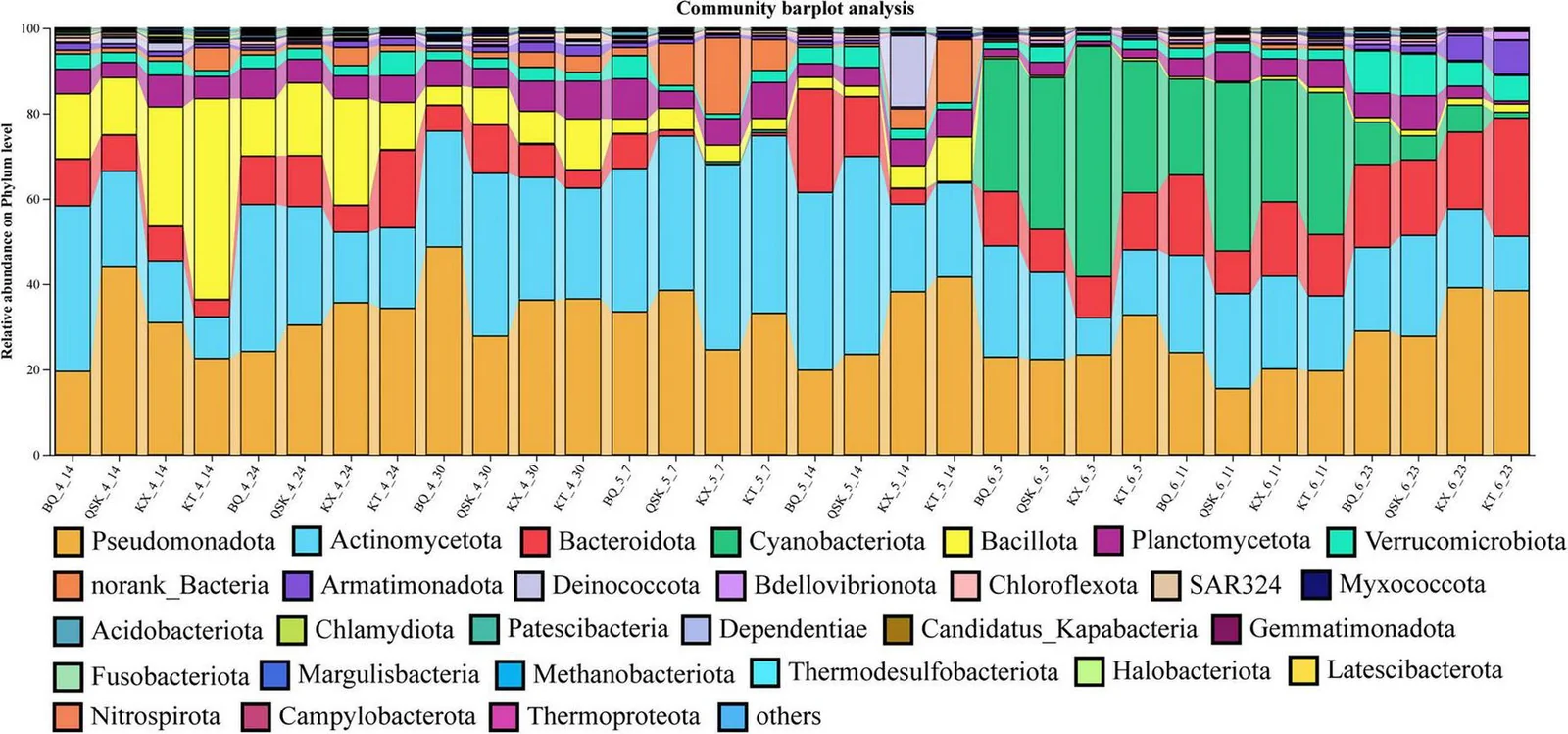
Temporal variation in the phylum-level composition of surface-water bacterial communities in Guanchang Reservoir.

Class-level composition reflected the same temporal reorganization. *Gammaproteobacteria* and *Actinobacteria* represented persistent and abundant components across much of the time series, together with *Bacteroidia*, *Alphaproteobacteria*, *Acidimicrobiia*, *Clostridia*, *Planctomycetes*, and other classes. *Clostridia* contributed more strongly to several early sampling communities and subsequently declined. In contrast, *Cyanobacteriia* increased substantially during the early-June sampling events, corresponding closely to the increase in *Cyanobacteriota* observed at the phylum level (Supplementary Figure 2). Family-level profiles provided further resolution of these temporal patterns. *Sporichthyaceae*, *Cyanobiaceae*, *Comamonadaceae*, *Ilumatobacteraceae*, *Moraxellaceae*, *Chitinophagaceae*, *Gemmataceae*, *Burkholderiaceae*, *Clostridiaceae*, *Peptostreptococcaceae*, and *Erysipelotrichaceae* represented important components of the bacterial assemblage. *Cyanobiaceae* increased conspicuously during early June and accounted for much of the broader cyanobacterial increase. Conversely, *Clostridiaceae*, *Peptostreptococcaceae*, and *Erysipelotrichaceae* were more strongly represented during the earlier portion of the time series. *Comamonadaceae* and several other families contributed more strongly again toward the later sampling period (Supplementary Figure 3).

The genus-level composition revealed the clearest temporal replacement among dominant bacterial populations. Major or recurrent genera included *Cyanobium* PCC-6307, hgcI clade, unclassified *Comamonadaceae*, CL500-29 marine group, *Enhydrobacter*, unclassified *Sporichthyaceae*, norank *Gemmataceae*, *Polynucleobacter*, *Clostridium*, *Sediminibacterium*, *Turicibacter*, *Terrisporobacter*, *Flavobacterium*, unclassified *Actinobacteria*, norank *Hyphomicrobiales*, *Chthoniobacter*, and *Fluviicola*. Several of these taxa exhibited contrasting temporal trajectories. *Clostridium*, *Turicibacter*, and *Terrisporobacter* contributed relatively large proportions to several of the earliest April samples but declined progressively through May and were present at much lower relative abundances during June. *Polynucleobacter* and CL500-29 marine group similarly showed stronger representation during portions of the spring sampling period and became less prominent during the early-June assemblages.

In contrast, *Cyanobium* PCC-6307 showed the most pronounced increase of any dominant genus. It remained comparatively rare during most April and May samples but increased sharply on 5 June across all four sampling stations. Its relative abundance remained high on 11 June, producing a conspicuous *Cyanobium*-rich community state across the reservoir. By 23 June, however, its relative abundance had decreased substantially, demonstrating that the early-June increase was transient rather than a monotonic increase throughout summer. Several heterotrophic bacterial groups increased again during the final sampling period. Unclassified *Comamonadaceae* became more prominent in late June, while other *Actinomycetota*- and *Pseudomonadota*-related taxa also contributed increasingly to the 23 June communities. As a result, late-June bacterial assemblages differed not only from the spring communities but also from the *Cyanobium*-rich assemblages observed on 5 and 11 June (Figure 5).

**FIGURE 5:**
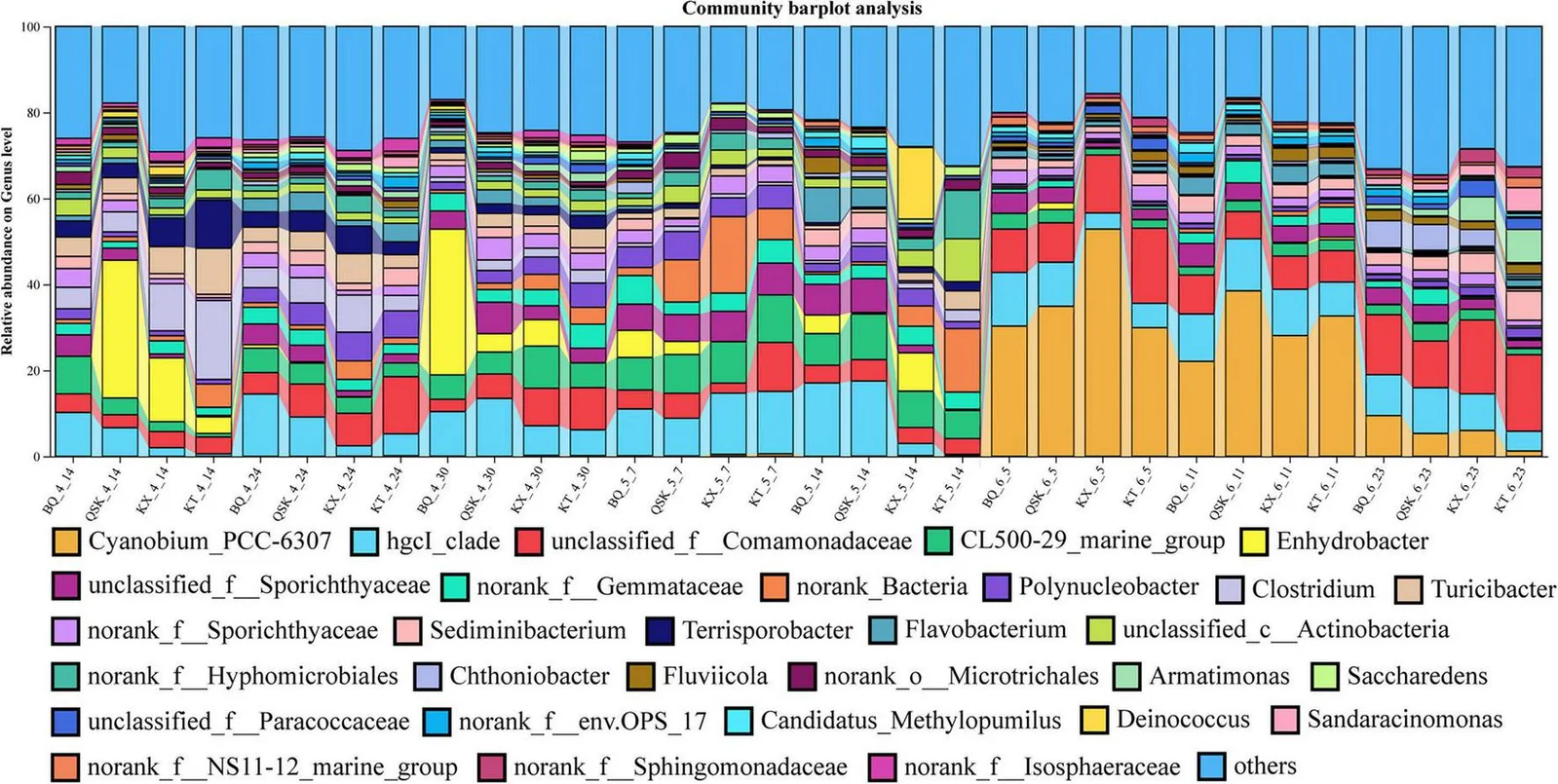
Temporal variation in the genus-level composition of surface-water bacterial communities in Guanchang Reservoir.

Collectively, the taxonomic profiles indicate that short-term temporal succession was characterized by persistence of several major freshwater bacterial phyla accompanied by substantial turnover at finer taxonomic levels. The spring-to-early-summer transition involved declining contributions of several taxa associated with the earlier sampling period, a transient increase in the *Cyanobium*-affiliated lineage across all four sampled stations, and a subsequent redistribution of dominance among multiple heterotrophic bacterial groups by late June.

### 3.5 Temporal restructuring of dominant ASVs

Fine-scale ASV composition further supported the temporal restructuring identified by NMDS and taxonomic profiling. The heatmap of the 50 most abundant ASVs showed clear temporal clustering, with several ASVs displaying greater relative abundance during April and May and a different subset becoming prominent during early June. The 23 June samples showed a further rearrangement rather than persistence of the early-June pattern (Figure 6). ASV922 was among the most conspicuous early-summer-associated ASVs, increasing markedly on 5 and 11 June, whereas ASV71 showed greater representation during earlier sampling periods. Taxonomic affiliations of the dominant ASVs highlighted here are provided in Supplementary Table 1. Overall, the ASV-level patterns demonstrate that the temporal differences observed at broader taxonomic levels were accompanied by turnover among dominant sequence variants.

**FIGURE 6:**
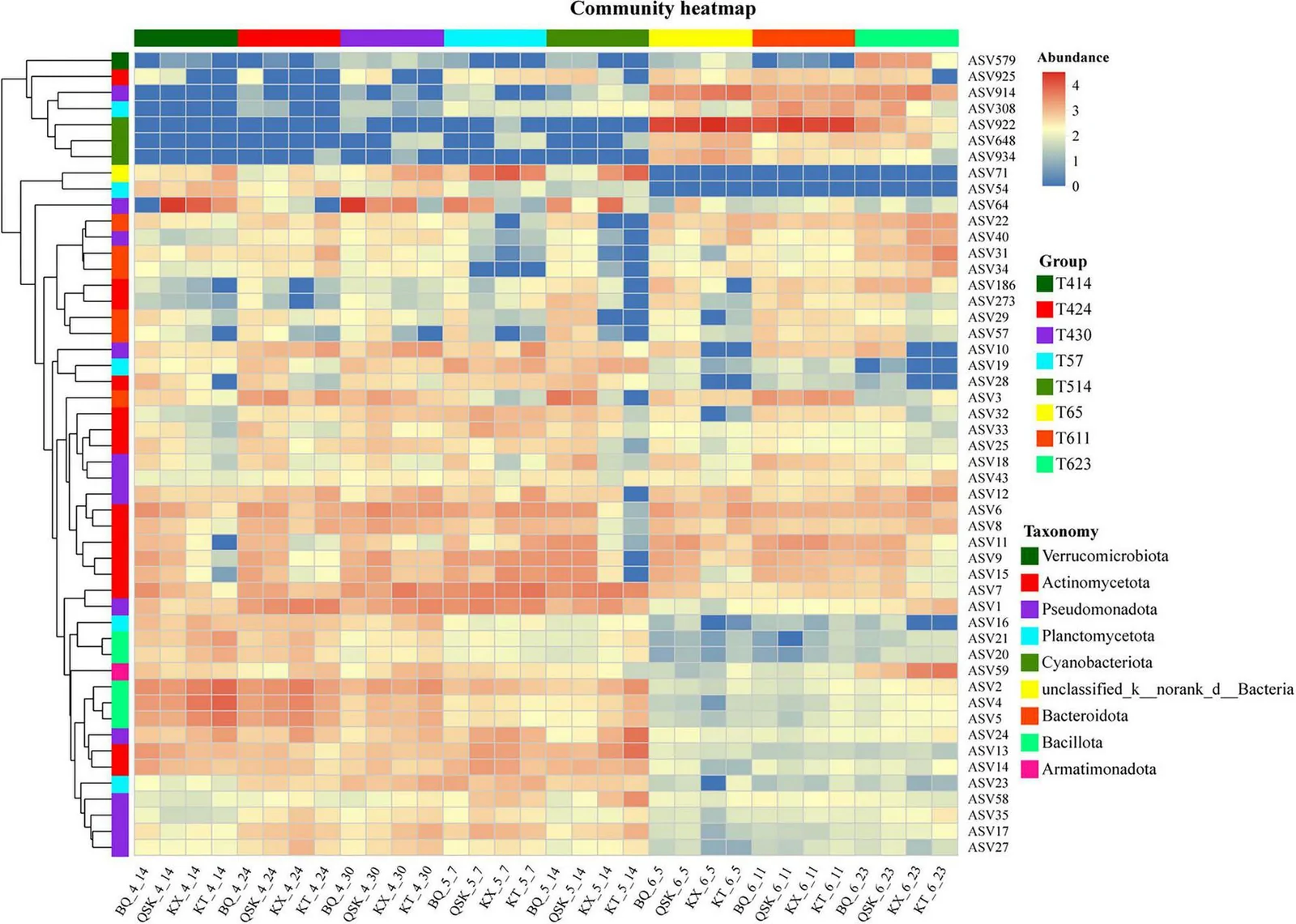
Temporal distribution of the 50 most abundant bacterial ASVs in Guanchang Reservoir. Heatmap showing abundance patterns of the 50 most abundant ASVs across the 32 surface-water samples. Columns represent individual samples arranged according to sampling date, whereas rows represent individual ASVs. The side annotation indicates the phylum-level taxonomic affiliation of each ASV.

Thus, the major temporal trajectory observed at the community level was reproduced at fine taxonomic resolution. The coordinated changes among dominant ASVs provide additional evidence that the beta-diversity pattern reflected substantial restructuring of bacterial community composition rather than variation confined to low-abundance taxa.

### 3.6 Differential bacterial taxa across sampling dates

Kruskal–Wallis analyses identified significant temporal differences at family, genus, and ASV levels (Figure 7). At the family level, significant differences were detected for *Cyanobiaceae* (*P* = 0.00034), *Erysipelotrichaceae* (*P* = 0.00030), *Peptostreptococcaceae* (*P* = 0.00016), *Clostridiaceae* (*P* = 0.00015), *Burkholderiaceae* (*P* = 0.00037), *Gemmataceae* (*P* = 0.00901), *Chitinophagaceae* (*P* = 0.01609), *Ilumatobacteraceae* (*P* = 0.00299), *Comamonadaceae* (*P* = 0.00369), and *Hyphomicrobiales*-related taxa (*P* = 0.00481).

**FIGURE 7:**
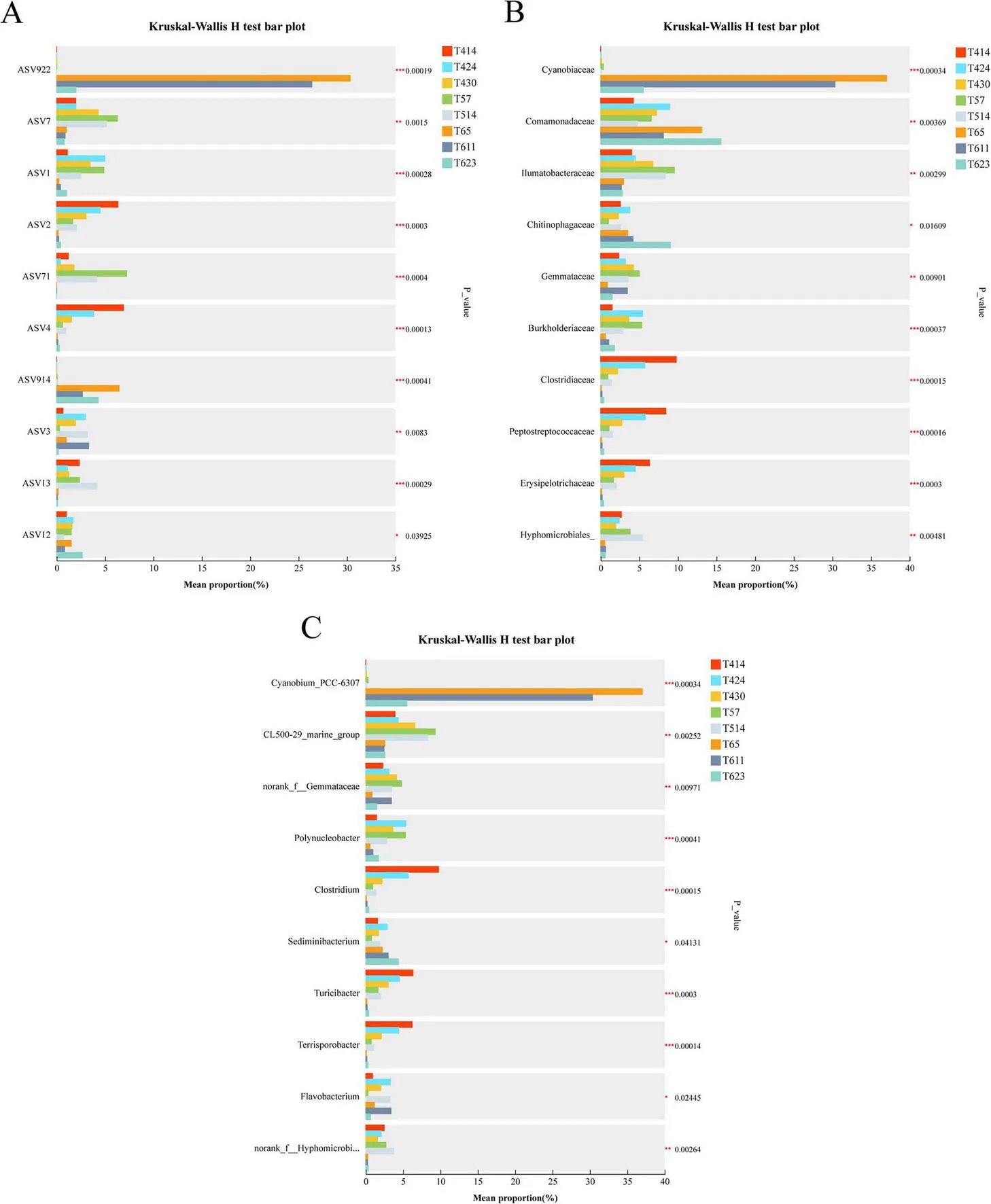
Bacterial taxa showing significant temporal variation across the spring-to-early-summer transition in Guanchang Reservoir. Kruskal–Wallis H tests were used to identify bacterial taxa whose relative abundances differed significantly among the eight sampling dates at the panels **(A)** ASV, **(B)** family, and **(C)** genus levels. Bars represent the mean relative abundance of each taxon within each sampling-date group. Asterisks indicate statistical significance: *P < 0.05, **P < 0.01, and ***P < 0.001.

At the genus level, significant temporal variation was observed for the *Cyanobium*-affiliated lineage (*P* = 0.00034), *Clostridium* (*P* = 0.00015), *Turicibacter* (*P* = 0.00030), *Terrisporobacter* (*P* = 0.00014), *Polynucleobacter* (*P* = 0.00041), the CL500-29 marine group (*P* = 0.00252), norank *Gemmataceae* (*P* = 0.00971), *Sediminibacterium* (*P* = 0.04131), *Flavobacterium* (*P* = 0.02445), and norank *Hyphomicrobiales* (*P* = 0.00264).

At the ASV level, significant temporal differences were detected for all 10 highlighted ASVs: ASV4 (*P* = 0.00013), ASV922 (*P* = 0.00019), ASV1 (*P* = 0.00028), ASV13 (*P* = 0.00029), ASV2 (*P* = 0.00030), ASV71 (*P* = 0.00040), ASV914 (*P* = 0.00041), ASV7 (*P* = 0.00150), ASV3 (*P* = 0.00830), and ASV12 (*P* = 0.03925). These statistical comparisons support the temporal differentiation identified independently from the community-composition and ASV-level analyses.

Overall, the differential-abundance analyses were consistent across ASV, family, and genus levels. The spring-to-early-summer transition involved opposing temporal trajectories between taxa that were enriched during the earlier spring period and taxa that became prominent during early summer, particularly *Cyanobium* PCC-6307.

### 3.7 Environmental associations with bacterial succession

Canonical correspondence analysis demonstrated clear relationships between bacterial community composition and the measured physicochemical variables. CCA1 explained 18.16% of community variation and CCA2 explained an additional 7.89%, giving a combined contribution of 26.05% for the first two constrained axes. The orientation of environmental vectors corresponded closely with the observed temporal succession. Temperature was directed predominantly toward the region occupied by later sampling communities, particularly the June samples. DO showed a broadly similar orientation. In contrast, TN and TP were directed toward the region associated with earlier spring communities, reflecting their higher concentrations during April. NH_4_^+^-N also contributed to separation toward earlier sampling conditions, whereas pH contributed additional variation along the constrained ordination space (Figure 8). The CCA pattern therefore paralleled the temporal environmental trends observed in the reservoir. Early communities were associated with comparatively higher TN and TP, whereas later communities were increasingly associated with warmer conditions. The ordination further indicates that community restructuring occurred along multiple correlated environmental gradients rather than along a single physicochemical variable.

**FIGURE 8:**
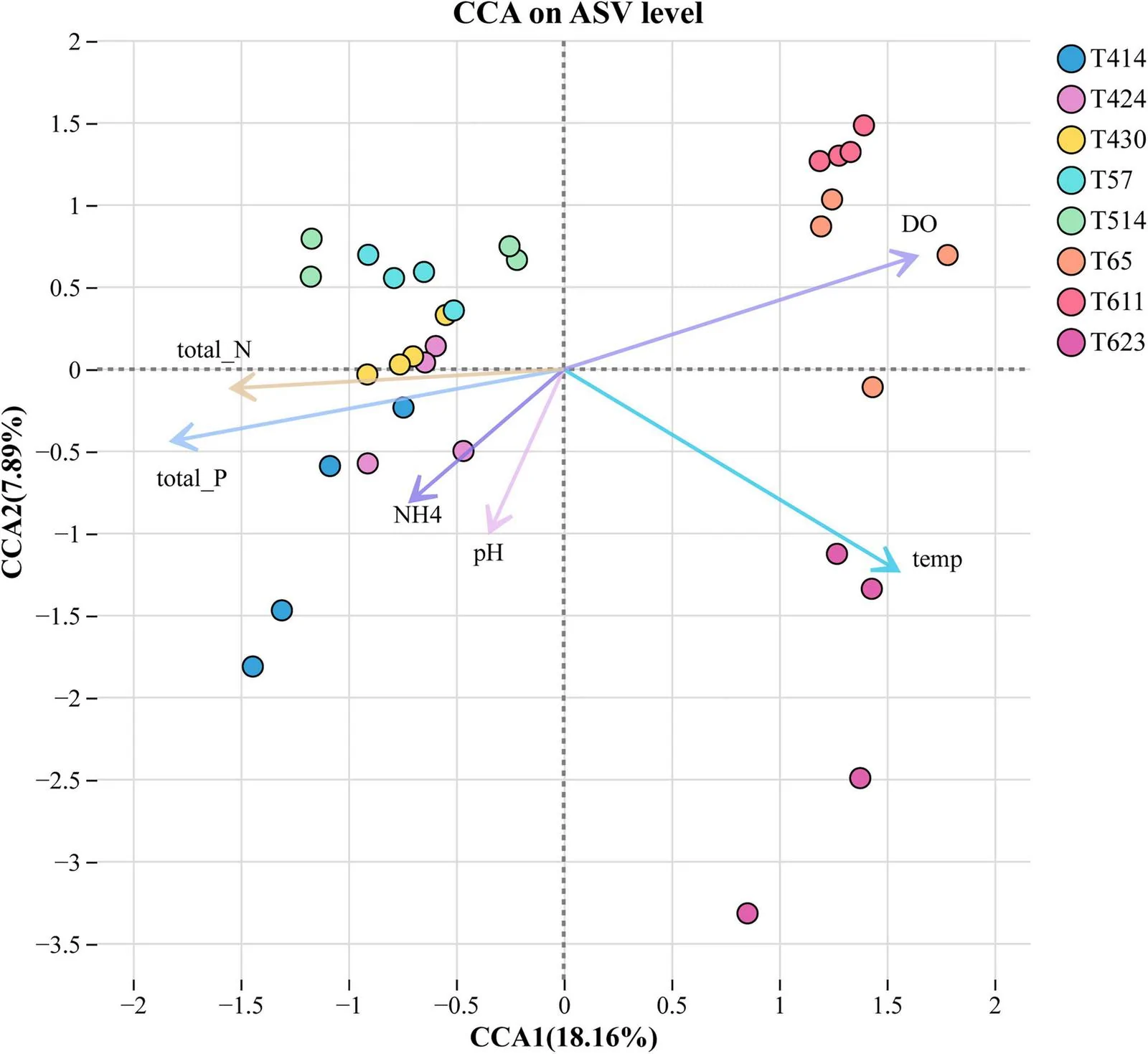
Relationships between surface-water bacterial community composition and environmental variables in Guanchang Reservoir. Canonical correspondence analysis (CCA) of bacterial communities at the ASV level in relation to water temperature, pH, dissolved oxygen (DO), ammonium nitrogen (NH_4_^+^-N), total phosphorus (TP), and total nitrogen (TN). Points represent individual bacterial community samples, with colors indicating the eight sampling dates. Arrows indicate the direction of associations between environmental variables and bacterial community composition.

Genus-level Spearman correlations provided additional resolution of these relationships. *Cyanobium* PCC-6307 was positively correlated with temperature (*r* = 0.489) and DO (*r* = 0.583), but negatively correlated with NH_4_^+^-N (*r* = −0.517), TP (*r* = −0.727), and TN (*r* = −0.746). Thus, the pronounced early-June increase in *Cyanobium* occurred during a period characterized by warmer conditions and substantially lower bulk nutrient concentrations than those observed during the earliest spring sampling. Unclassified *Comamonadaceae* showed a similar relationship with nutrient concentrations, with strong negative correlations with TP (*r* = −0.647) and especially TN (*r* = −0.818). *Fluviicola* was positively associated with temperature (*r* = 0.584) and negatively associated with TP (*r* = −0.631) and TN (*r* = −0.461). *Sediminibacterium* also showed negative relationships with TP (*r* = −0.590) and TN (*r* = −0.467).

In contrast, several taxa associated with earlier sampling periods showed the opposite environmental pattern. Clostridium was negatively correlated with temperature (*r* = −0.496) and DO (*r* = −0.620), but positively correlated with NH_4_^+^-N (*r* = 0.470), TP (*r* = 0.808), and TN (*r* = 0.672). *Turicibacter* showed an almost identical pattern, with negative relationships with temperature (*r* = −0.496) and DO (*r* = −0.621) and strong positive correlations with TP (*r* = 0.809) and TN (*r* = 0.668). *Terrisporobacter* was likewise negatively associated with temperature (*r* = −0.494) and DO (*r* = −0.636), while showing positive relationships with NH_4_^+^-N (*r* = 0.471), TP (*r* = 0.796), and TN (*r* = 0.677).

The similarity of these three correlation profiles was striking and corresponded closely with their shared temporal decline from spring toward early summer. Other spring-associated taxa also exhibited related patterns. CL500-29 marine group was negatively correlated with temperature (*r* = −0.584) and positively associated with TP (*r* = 0.384) and TN (*r* = 0.386). *Polynucleobacter* showed negative correlations with temperature (*r* = −0.615) and DO (*r* = −0.559), whereas norank *Microtrichales* was negatively associated with temperature and DO but positively associated with TP and TN (Figure 9).

**FIGURE 9:**
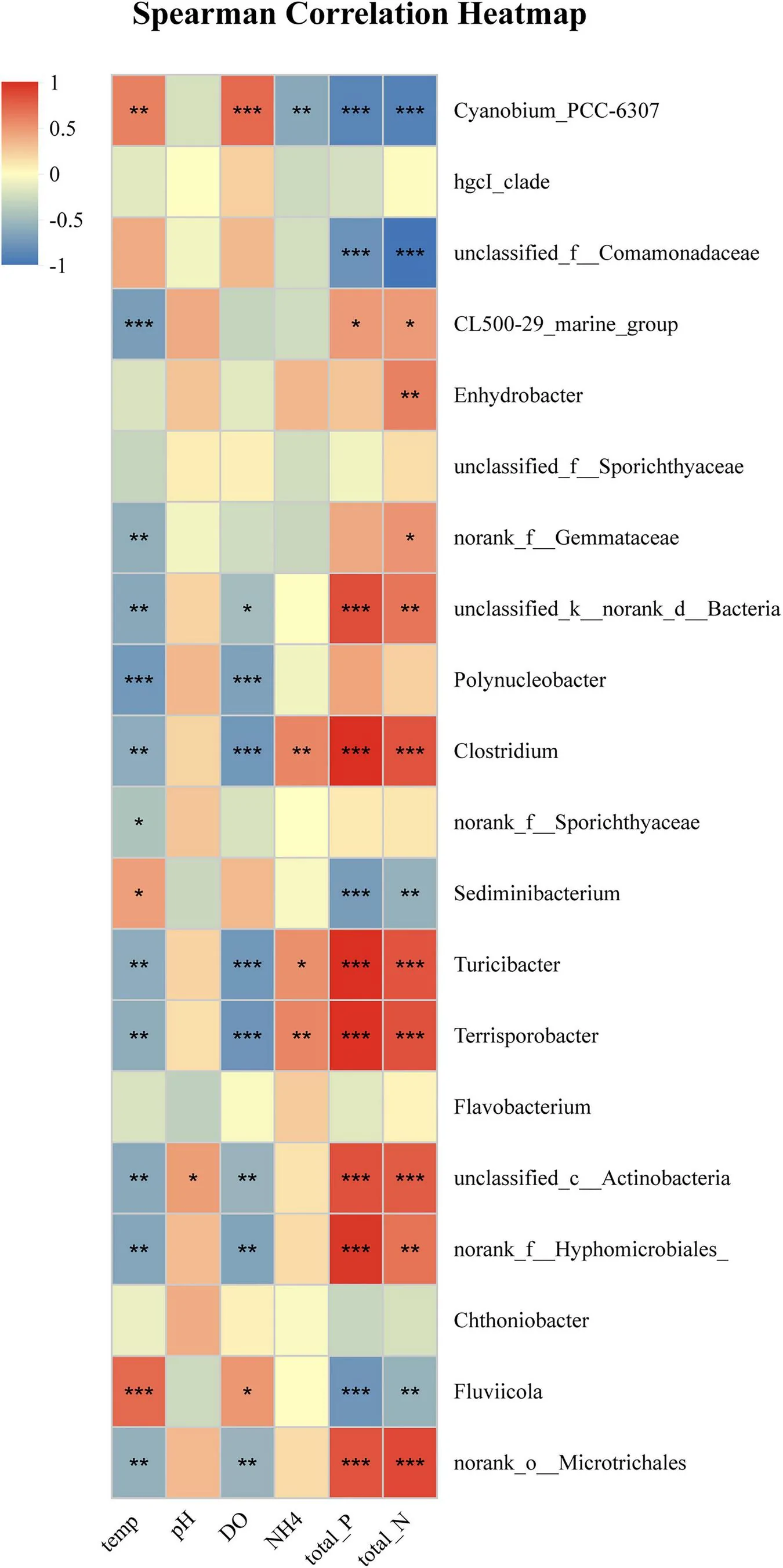
Spearman correlations between dominant bacterial genera and environmental variables in Guanchang Reservoir. Heatmap showing Spearman correlation coefficients between dominant bacterial genera and water temperature, pH, dissolved oxygen (DO), ammonium nitrogen (NH_4_^+^-N), total phosphorus (TP), and total nitrogen (TN). Cell colors indicate the direction and magnitude of the correlation coefficients, with red representing positive correlations and blue representing negative correlations. Asterisks indicate statistical significance: *P < 0.05, **P < 0.01, and ***P < 0.001.

Together, the CCA and genus-level correlations revealed two contrasting environmental association patterns. Several taxa characteristic of the spring assemblage were associated with cooler conditions and comparatively higher nitrogen and phosphorus concentrations, whereas taxa enriched during the later portion of the study, including *Cyanobium*, unclassified *Comamonadaceae*, *Fluviicola*, and *Sediminibacterium*, tended to show stronger associations with warmer conditions and/or lower nutrient concentrations. These opposing relationships provide environmental context for the taxonomic replacement observed across the spring-to-early-summer transition.

## 4 Discussion

### 4.1 Pronounced bacterial succession during the spring-to-early-summer transition

The most prominent ecological pattern in Guanchang Reservoir was the pronounced temporal reorganization of the surface-water bacterial community during the approximately 10-week transition from mid-April to late June. Significant changes in richness and Shannon diversity were accompanied by strong temporal differentiation in overall community structure. More importantly, the NMDS ordination suggested a directional progression from April assemblages through intermediate May communities to distinct early- and late-June states. This temporal organization indicates that bacterial communities did not simply fluctuate stochastically but underwent recognizable succession. Pronounced temporal turnover is a characteristic feature of freshwater bacterioplankton. Although freshwater ecosystems often retain broadly similar dominant bacterial phyla through time, stronger temporal variability generally emerges at finer taxonomic resolutions (Boucher et al., 2006; Newton et al., 2011). Long-term observations have demonstrated repeatable bacterial phenology in lakes, with seasonal community transitions associated with temperature, nutrient availability, mixing conditions, and biological interactions (Shade et al., 2007; Kara et al., 2013; Yan et al., 2017).

Reservoir bacterioplankton communities can undergo pronounced seasonal succession, with temporal turnover occurring across abundant, rare, and conditionally rare community fractions (Li et al., 2016; Nyirabuhoro et al., 2020). In Danjiangkou Reservoir, bacterial community composition and ecological characteristics varied markedly among seasons and were associated with environmental gradients including nitrogen and phosphorus (Chen et al., 2022). Seasonal restructuring has also been documented in plateau and tropical drinking-water reservoirs (Wu et al., 2024; Yang et al., 2024).

The present study resolves this succession at a comparatively fine temporal scale. Rather than sampling only broadly separated seasons, eight sampling events were concentrated within one spring-to-early-summer transition. The resulting patterns show that substantial microbial reorganization occurred over intervals of only weeks. Similar short-term bacterial turnover has been reported in freshwater systems where temporal beta diversity develops rapidly under changing environmental conditions (Jones et al., 2012; Paruch et al., 2020). The distinction between broad seasonal states and short-lived transitional states is therefore important. Guanchang Reservoir did not simply shift from one stable spring assemblage to a single summer assemblage. Instead, May and early June represented intermediate states, and the early- and late-June assemblages themselves differed markedly. The spring-to-early-summer transition observed here therefore represented a dynamic sequence of community states rather than a simple contrast between two seasonal endpoints. Importantly, the repeated sampling design captured short-lived intermediate states, particularly the early-June community, that would not have been resolved by a conventional comparison based only on spring and summer endpoints.

### 4.2 Persistent freshwater bacterial lineages coexisted with rapid fine-scale turnover

Despite strong temporal succession, Guanchang Reservoir retained a broadly stable freshwater taxonomic framework. *Pseudomonadota*, *Actinomycetota*, *Bacteroidota*, *Cyanobacteriota*, *Planctomycetota*, *Bacillota*, and *Verrucomicrobiota* occurred throughout the study. These phyla are widely recognized as major components of freshwater bacterioplankton communities (Newton et al., 2011). The simultaneous occurrence of 195 ASVs across all eight sampling periods further indicates the presence of a persistent bacterial component. At the same time, large numbers of date-specific ASVs demonstrate that this persistent background was accompanied by a highly dynamic fraction. Such coexistence of persistent and temporally responsive populations has been observed in other freshwater environments, where a relatively stable microbial core can coexist with substantial turnover among more responsive community members (Jones et al., 2012; Nyirabuhoro et al., 2020).

Several recurrent taxa in Guanchang Reservoir are well-recognized freshwater bacterial lineages. The hgcI clade and CL500-29-related *Actinomycetota* occurred repeatedly across the time series. Freshwater *Actinomycetota* are frequently abundant in lakes and reservoirs and exhibit considerable ecological differentiation at finer phylogenetic levels (Allgaier and Grossart, 2006; Newton et al., 2007; Ghai et al., 2014). The significant temporal variation of CL500-29 therefore illustrates an important distinction between persistence and ecological stability: a lineage may remain detectable throughout a seasonal transition while its relative contribution changes substantially. *Polynucleobacter* similarly displayed pronounced temporal dynamics. Members of this genus are widespread in freshwater habitats and can exhibit highly predictable seasonal population patterns (Wu and Hahn, 2006). In Guanchang Reservoir, *Polynucleobacter* was relatively more prominent during the cooler portion of the sampling period and was negatively associated with temperature and DO, contributing to differentiation between spring and early-summer assemblages. A particularly coherent response was observed for *Clostridium*, *Turicibacter*, and *Terrisporobacter*. All three were more abundant during earlier sampling periods, declined toward June, and displayed similar environmental associations, including positive relationships with TP and TN and negative relationships with temperature and DO. Their parallel temporal behavior indicates that they formed part of a spring-associated bacterial assemblage that became progressively less represented as reservoir conditions shifted.

These taxa should nevertheless be interpreted primarily as indicators of contrasting temporal community states. Genus-level 16S rRNA gene assignments do not directly establish metabolic activity or ecological function, and taxonomically related strains may exhibit substantial functional heterogeneity.

### 4.3 Changing thermal and nutrient conditions were closely associated with microbial succession

The contrasting genus-level correlations observed in Guanchang Reservoir indicate that taxonomic turnover occurred concurrently with changes in the thermal environment. The *Cyanobium*-affiliated lineage and *Fluviicola* were positively associated with temperature, whereas CL500-29, *Polynucleobacter*, *Clostridium*, *Turicibacter*, and *Terrisporobacter* showed negative associations. These patterns are consistent with differential responses among bacterial populations along the observed environmental gradient, although the observational design does not allow individual environmental variables to be identified as independent causal drivers. Temperature is among the most consistently identified correlates of freshwater bacterioplankton seasonality. It can influence microbial growth, enzymatic activity, organic-matter processing, phytoplankton production, water-column stability, and trophic interactions (Newton et al., 2011). Seasonal bacterial studies repeatedly identify temperature as an important predictor of community composition in lakes and reservoirs (Shade et al., 2007; Yan et al., 2017; Paruch et al., 2020; Yang et al., 2024).

The contrasting genus-level correlations in Guanchang Reservoir are consistent with environmental sorting along the thermal gradient. *Cyanobium* PCC-6307 and *Fluviicola* were positively associated with temperature, whereas CL500-29, *Polynucleobacter*, *Clostridium*, *Turicibacter*, and *Terrisporobacter* showed negative relationships. Such contrasting responses indicate that warming was accompanied by replacement among bacterial populations occupying different ecological niches. Changes in nitrogen and phosphorus were equally conspicuous. *Clostridium*, *Turicibacter*, and *Terrisporobacter* were positively associated with TP and TN, whereas *Cyanobium* PCC-6307 and unclassified *Comamonadaceae* showed negative associations. Similar relationships between bacterioplankton composition and nitrogen or phosphorus availability have been reported in subtropical reservoirs (Li et al., 2016; Chen et al., 2022; Wu et al., 2024). The decline in bulk nutrient concentrations should nevertheless be interpreted within the broader spring-to-early-summer transition. TN and TP represent integrated ecosystem-state variables influenced by catchment inputs, biological uptake, remineralization, sediment exchange, and hydrological transport. Consequently, correlations between individual bacterial taxa and nutrients do not necessarily represent direct physiological responses to a single variable. Moreover, because sampling date, temperature, and nutrient concentrations changed concurrently, their independent effects cannot be disentangled from the present observational dataset.

Reservoir microbial communities are influenced by interacting environmental processes. Water temperature, nutrient retention, residence time, stratification, mixing, and hydrological connectivity can jointly influence microbial assembly (Lindström and Bergström, 2004;Lindström et al., 2005; Niño-García et al., 2016; Li et al., 2022). The observed bacterial succession in Guanchang Reservoir is therefore most consistently interpreted as community reorganization along an integrated spring-to-early-summer environmental gradient.

### 4.4 A transient *Cyanobium*-rich state marked the early-summer transition

The strongest taxonomic feature of the seasonal transition was the pronounced increase in *Cyanobium* PCC-6307 during 5 and 11 June. The same ecological event was evident at multiple taxonomic levels, including increases in *Cyanobacteriota*, *Cyanobacteriia*, *Cyanobiaceae*, and *Cyanobium*. The simultaneous response across all four sampling stations indicates that the early-June *Cyanobium*-rich state represented a broad surface-water phenomenon rather than an isolated local event. Freshwater picocyanobacteria are widespread across lakes and reservoirs and can become important components of plankton communities under a broad range of trophic conditions (Callieri, 2010). High-resolution molecular studies have demonstrated pronounced seasonal and vertical niche differentiation among *Cyanobium* populations (Nwosu et al., 2021; Carratalà et al., 2023). Temporal changes in *Cyanobium* can therefore provide an informative indicator of broader cyanobacterial community succession.

In Guanchang Reservoir, *Cyanobium* PCC-6307 was positively associated with temperature and DO but negatively associated with NH_4_^+^-N, TP, and TN. Its early-June increase consequently occurred under an environmental regime that differed substantially from that associated with the spring bacterial assemblage. Picocyanobacteria possess small cell sizes and high surface-area-to-volume ratios, which may enhance resource acquisition under some relatively nutrient-poor conditions (Callieri, 2010). These relationships should, however, be interpreted as temporal associations rather than direct physiological responses. Notably, the relative abundance of this lineage decreased markedly by 23 June even though surface-water temperature continued to increase, indicating that temperature alone cannot account for its temporal pattern. Other unmeasured biological, physicochemical, or hydrological factors may also have contributed to the observed dynamics.

The early-June increase is relevant to understanding cyanobacterial community succession but does not demonstrate the occurrence of a harmful cyanobacterial bloom. Relative abundance derived from 16S rRNA gene sequencing does not directly quantify cyanobacterial cell density, chlorophyll-a, phycocyanin, absolute biomass, or toxin production. The present result should therefore be interpreted as a transient compositional increase in a *Cyanobium*-affiliated lineage rather than as evidence of bloom formation. Temporal restriction of *Cyanobium* dominance has also been observed during cyanobacterial succession in other freshwater systems. Cyanobacterial communities may undergo marked succession, with individual taxa becoming dominant only during relatively short ecological phases (Li et al., 2020). The increase in *Cyanobium* is relevant to reservoir water-quality assessment but does not directly demonstrate a harmful cyanobacterial bloom. Cyanobacterial bloom development reflects complex interactions among nutrients, warming, water-column stability, residence time, and biological processes (Paerl and Otten, 2013; Paerl et al., 2016; Visser et al., 2016; Huisman et al., 2018; Smucker et al., 2021). Moreover, relative 16S rRNA gene sequence abundance does not directly measure cyanobacterial cell density, chlorophyll-a, phycocyanin, biomass, or toxin production.

The ecological significance of the present result lies in the rapid and spatially coherent emergence of a *Cyanobium*-rich bacterial state that persisted for only part of the early-summer transition. This transient phase demonstrates that ecologically important cyanobacterial changes can occur over periods substantially shorter than conventional seasonal sampling intervals.

### 4.5 Temporal coherence across the four sampled reservoir stations

Repeated sampling of the four fixed stations provided insight into the spatial consistency of the observed temporal changes within the sampled portions of the reservoir. Although station-specific differences were present, several major bacterial transitions occurred concurrently at multiple stations. The early-June increase in the *Cyanobium*-affiliated lineage was particularly notable because it was observed at the reservoir-head, intermediate, water-intake, and dam-front stations. This consistency indicates that the major temporal transition was not restricted to a single sampled location. However, because the present study included four fixed stations rather than comprehensive spatial coverage of the entire reservoir, these observations should not be interpreted as demonstrating spatial uniformity throughout Guanchang Reservoir (Li et al., 2016; Nyirabuhoro et al., 2020; Chen et al., 2022; Wu et al., 2024).

The ecological context of Guanchang Reservoir is fundamentally shaped by water-conservancy engineering. Damming transforms flowing-water environments into systems characterized by lower current velocity, increased retention time, altered connectivity, enhanced sedimentation, and modified nutrient and thermal conditions. These changes influence both microbial dispersal and environmental selection (Lindström et al., 2006; Niño-García et al., 2016). Studies of dam-affected systems have demonstrated substantial microbial responses to this engineering context. Dam construction altered bacterial community structure in riparian sediments of the Lancang River (Chen et al., 2018), changed sulfur-associated microbial assemblages in Xiaowan Reservoir (Shi et al., 2020), and modified taxonomic and functional organization of bacterioplankton across cascade reservoirs (Li et al., 2022; Jiang et al., 2023). Changes in planktonic microbial food-web structure have likewise been observed in dam-impacted reaches of the Yangtze River (Wang et al., 2023). Guanchang Reservoir can therefore be considered a water-conservancy-regulated ecological system within which seasonal microbial succession occurs. The engineering setting establishes the physical framework of the ecosystem, whereas seasonal environmental variation continuously reorganizes microbial populations within that framework.

The QSK station was located near a bottom-aeration system used for local water-quality management. However, the present sampling design did not include pre-aeration observations or replicated aerated and non-aerated controls. The QSK station was therefore treated only as one of the four fixed spatial sampling locations, and no causal effect of bottom aeration on the bacterial community was evaluated. Reservoir studies have demonstrated strong depth-dependent microbial differentiation associated with thermal stratification and oxygen gradients (Zhang et al., 2015; Carratalà et al., 2023). The local aeration system should therefore be understood as one component of spatial environmental heterogeneity embedded within broader reservoir-scale seasonal succession.

### 4.6 Transient microbial states emphasize the ecological importance of seasonal transition periods

The eight-date time series revealed that microbial succession in Guanchang Reservoir involved a sequence of transient ecological states rather than a smooth transition between two stable seasonal endpoints. The early-June *Cyanobium*-rich community provides the clearest example. It coincided with reduced Shannon diversity and major restructuring of overall bacterial composition but had largely disappeared by 23 June. Such transient states are ecologically important because microbial communities can respond rapidly to environmental change. Short generation times, high dispersal potential, and rapid physiological responses allow bacterial assemblages to reorganize over periods considerably shorter than conventional seasonal monitoring intervals (Jones et al., 2012; Paruch et al., 2020).

Temporal resolution therefore strongly influences how reservoir microbial dynamics are interpreted. Sampling only at the beginning and end of the study period would have missed the pronounced early-June community state and produced a substantially simplified description of spring-to-summer succession. This issue is particularly relevant for cyanobacterial dynamics because different cyanobacterial populations can dominate during different stages of the warm season (Li et al., 2020; Nwosu et al., 2021; Carratalà et al., 2023). Repeated sampling during transitional periods can therefore reveal ecological events that are poorly represented by single seasonal observations.

The present results also identify several dimensions for future investigation. Vertical microbial profiling would determine whether the surface-water succession documented here extends into deeper portions of the reservoir. Incorporating reservoir water level, inflow, outflow, rainfall, hydraulic residence time, and mixing measurements would further clarify how seasonal environmental variation interacts with hydrological regulation. Integration of amplicon sequencing with chlorophyll-a, phycocyanin, cyanobacterial cell abundance, quantitative PCR, and metagenomic or metatranscriptomic analyses would additionally help distinguish changes in relative microbial composition from changes in absolute biomass and ecological activity. Overall, the convergence of alpha-diversity variation, NMDS differentiation, dominant-ASV turnover, taxonomic replacement, and environmental associations demonstrates that the spring-to-early-summer transition represents a period of substantial microbial reorganization in Guanchang Reservoir. The bacterial assemblage was therefore not a static component of reservoir water quality but a dynamic ecological community responding rapidly to changing environmental conditions within a water-conservancy-regulated ecosystem.

## 5 Conclusion

Surface-water bacterial communities in Guanchang Reservoir underwent pronounced and non-linear succession during the spring-to-early-summer transition. Significant temporal changes in bacterial richness and diversity were accompanied by extensive restructuring of community composition and replacement among dominant bacterial populations. Although major freshwater phyla persisted throughout the study, stronger differentiation occurred at genus and ASV levels. Spring assemblages contained relatively greater contributions from *Clostridium*, *Turicibacter*, *Terrisporobacter*, *Polynucleobacter*, and the CL500-29 marine group, whereas early June was characterized by a pronounced increase in *Cyanobacteriota* and the *Cyanobium*-affiliated lineage that was observed across all four sampled stations. This *Cyanobium*-rich state subsequently declined by late June, demonstrating that early-summer succession involved transient microbial states rather than a stable endpoint. Community restructuring occurred concurrently with changes in temperature and nutrient conditions, and contrasting associations among dominant taxa provided environmental context for the observed spring-to-early-summer community turnover. Collectively, these findings demonstrate that bacterial communities in a water-conservancy reservoir can reorganize rapidly within a relatively short spring-to-early-summer transition and highlight the ecological value of temporally resolved microbial monitoring for resolving short-lived community states.

## Data Availability

The datasets presented in this study can be found in online repositories. The names of the repository/repositories and accession number(s) can be found below: https://ngdc.cncb.ac.cn/gsa/search?searchTerm=CRA049302.

## Footnotes

1
https://ngdc.cncb.ac.cn/gsa/search?searchTerm=CRA049302

## Supplementary material

Supplementary Figure 1 Shared and sampling-date-specific bacterial taxa across the spring-to-early-summer transition in Guanchang Reservoir. Venn diagrams showing the numbers of shared and sampling-date-specific bacterial taxa across the eight sampling dates at the panels **(A)** phylum, **(B)** family, **(C)** genus, and **(D)** ASV levels. Numbers in the overlapping regions indicate taxa shared among sampling-date groups, whereas numbers in the non-overlapping regions indicate taxa uniquely detected at individual sampling dates. At the ASV level, 195 ASVs were shared across all eight sampling periods, while substantial numbers of date-specific ASVs were also detected.

Supplementary Figure 2 Temporal variation in the class-level composition of surface-water bacterial communities in Guanchang Reservoir.

Supplementary Figure 3 Temporal variation in the family-level composition of surface-water bacterial communities in Guanchang Reservoir.
