# Supplementary material for: Rapid restructuring of surface-water bacterial communities across the spring-to-early-summer transition in a water-conservancy reservoir
Source: Front Microbiol. 2026 Oct 8;17:1992256. doi: 10.3389/fmicb.2026.1992256 (PMC13687416; doi:10.3389/fmicb.2026.1992256)
Supplement: Supplementary file 1 [file Data_Sheet_1.zip › Supplementary Figure 3. Temporal variation in the family-level composition of surface-water bacterial communities in Guanchang Reservoir..pdf]

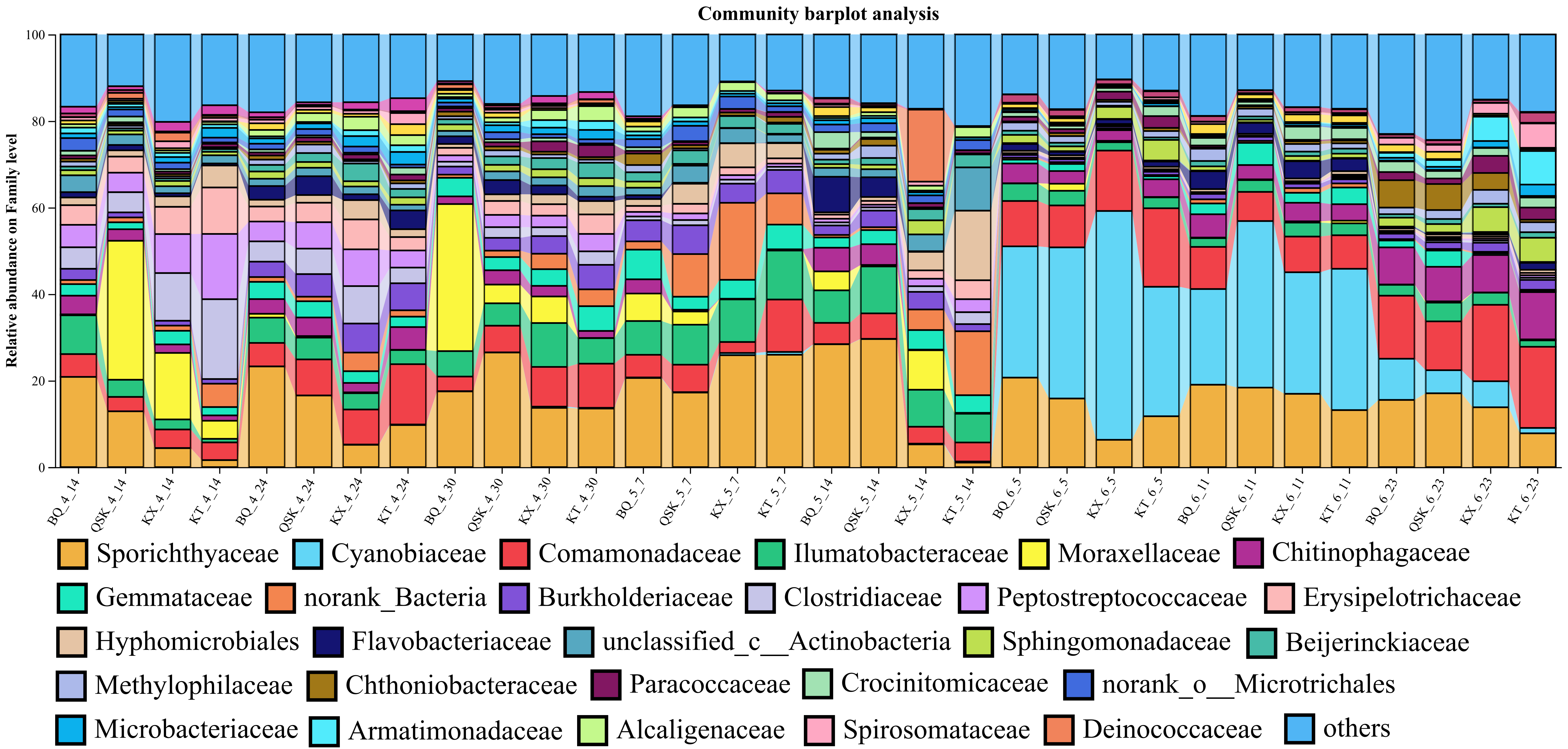
Community barplot analysis
Relative abundance on Family level
0
20
40
60
80
100
BQ_4_14
QSK_4_14
KX_4_14
KT_4_14
BQ_4_24
QSK_4_24
KX_4_24
KT_4_24
BQ_4_30
QSK_4_30
KX_4_30
KT_4_30
BQ_5_7
QSK_5_7
KX_5_7
KT_5_7
BQ_5_14
QSK_5_14
KX_5_14
KT_5_14
BQ_6_5
QSK_6_5
KX_6_5
KT_6_5
BQ_6_11
QSK_6_11
KX_6_11
KT_6_11
BQ_6_23
QSK_6_23
KX_6_23
KT_6_23
Sporichthyaceae
Cyanobiaceae
Comamonadaceae
Ilumatobacteraceae
Moraxellaceae
Chitinophagaceae
Gemmataceae
norank_Bacteria
Burkholderiaceae
Clostridiaceae
Peptostreptococcaceae
Erysipelotrichaceae
Hyphomicrobiales
Flavobacteriaceae
unclassified_c__Actinobacteria
Sphingomonadaceae
Beijerinckiaceae
Methylophilaceae
Chthoniobacteraceae
Paracoccaceae
Crocinitomicaceae
norank_o__Microtrichales
Microbacteriaceae
Armatimonadaceae
Alcaligenaceae
Spirosomataceae
Deinococcaceae
others
