# Supplementary material for: Rapid restructuring of surface-water bacterial communities across the spring-to-early-summer transition in a water-conservancy reservoir
Source: Front Microbiol. 2026 Oct 8;17:1992256. doi: 10.3389/fmicb.2026.1992256 (PMC13687416; doi:10.3389/fmicb.2026.1992256)
Supplement: Supplementary file 1 [file Data_Sheet_1.zip › Supplementary Figure 1. Shared and sampling-date-specific bacterial taxa across the spring-to-early-summer transition in Guanchang Reservoir..pdf]

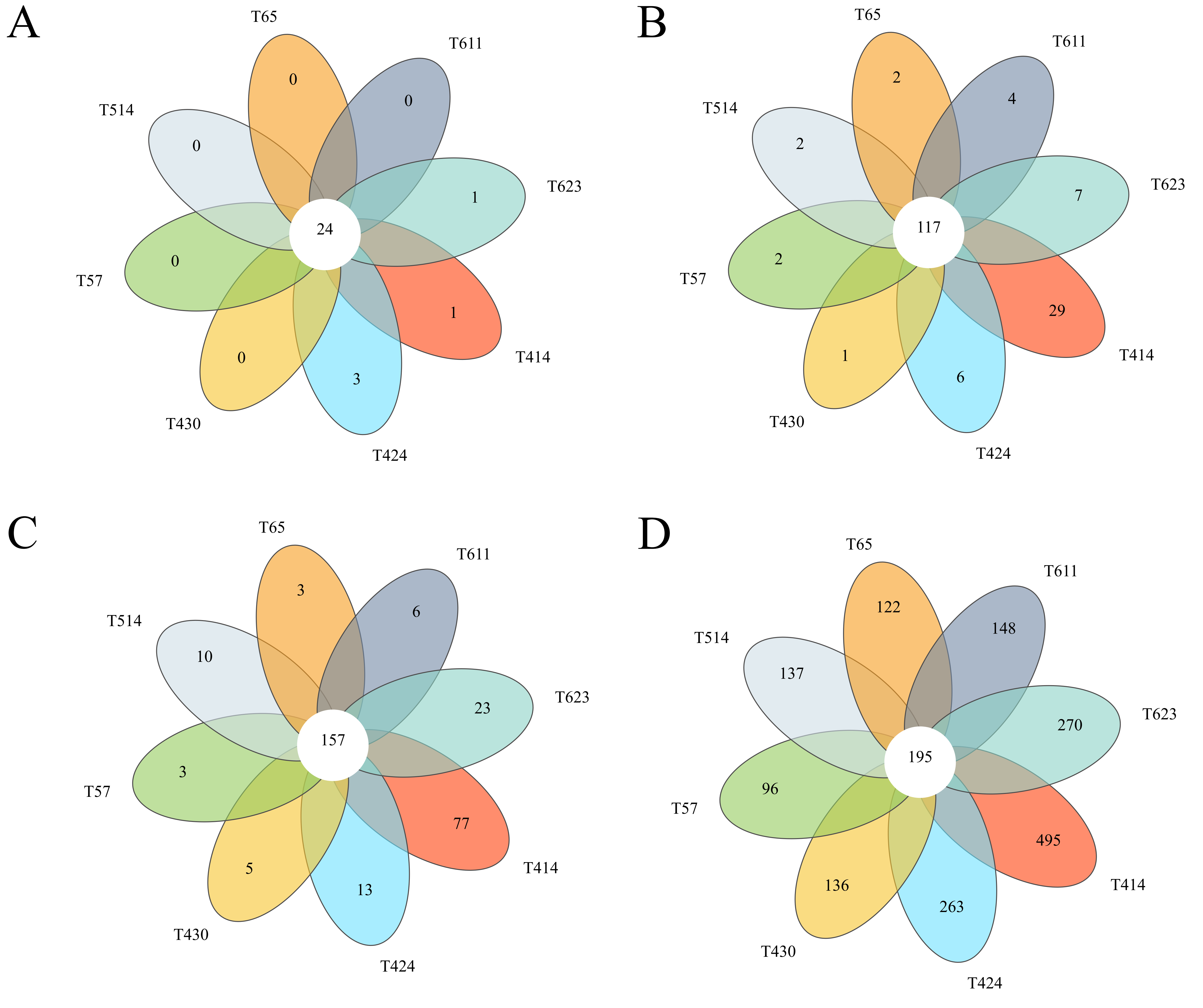
A
T65
T611
T623
T414
T424
T430
T57
T514
0
0
1
1
3
0
0
0
24
B
T65
T611
T623
T414
T424
T430
T57
T514
2
4
7
29
6
1
2
2
117
C
T65
T611
T623
T414
T424
T430
T57
T514
3
6
23
77
13
5
3
10
157
D
T65
T611
T623
T414
T424
T430
T57
T514
122
148
270
495
263
136
96
137
195
