# Supplementary material for: Rapid restructuring of surface-water bacterial communities across the spring-to-early-summer transition in a water-conservancy reservoir
Source: Front Microbiol. 2026 Oct 8;17:1992256. doi: 10.3389/fmicb.2026.1992256 (PMC13687416; doi:10.3389/fmicb.2026.1992256)
Supplement: Supplementary file 1 [file Data_Sheet_1.zip › Supplementary Figure 2. Temporal variation in the class-level composition of surface-water bacterial communities in Guanchang Reservoir..pdf]

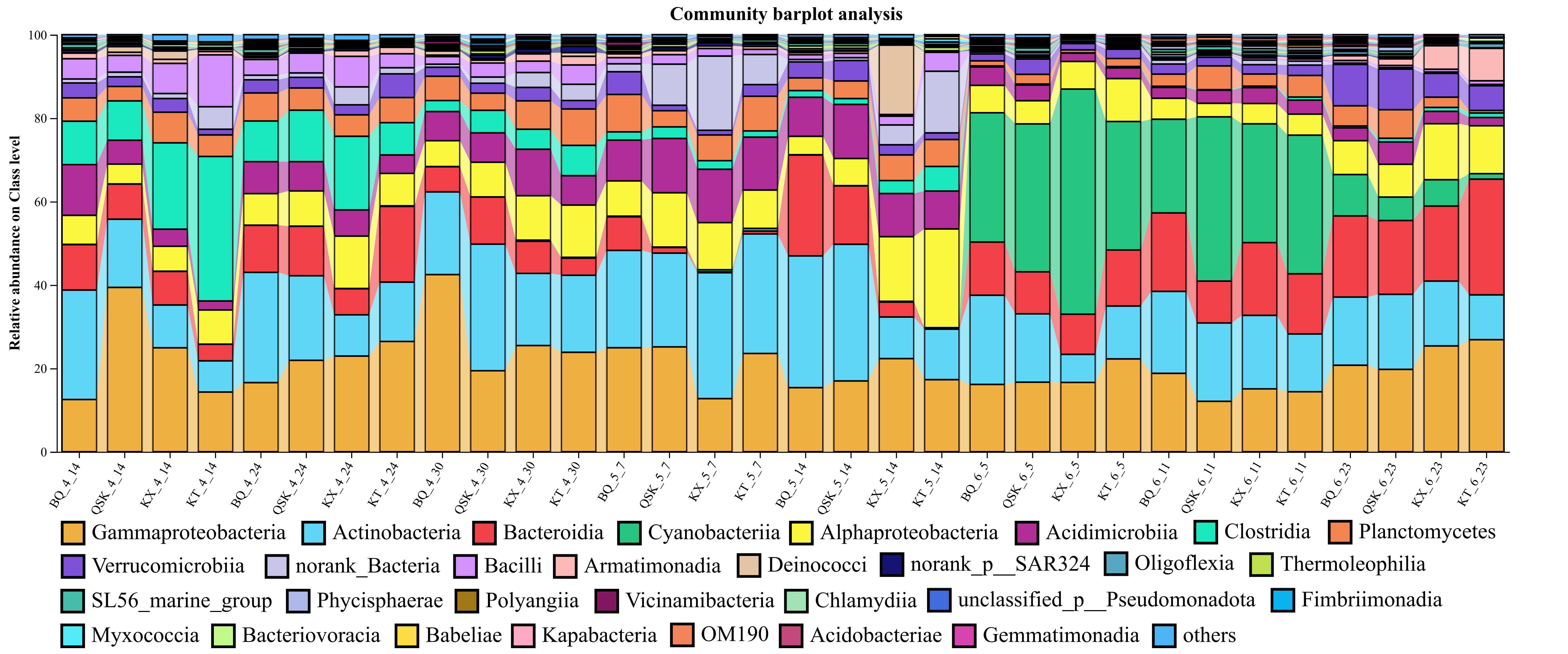

Community barplot analysis
Relative abundance on Class level
0
20
40
60
80
100
BQ_4_14
QSK_4_14
KX_4_14
KT_4_14
BQ_4_24
QSK_4_24
KX_4_24
KT_4_24
BQ_4_30
QSK_4_30
KX_4_30
KT_4_30
BQ_5_7
QSK_5_7
KX_5_7
KT_5_7
BQ_5_14
QSK_5_14
KX_5_14
KT_5_14
BQ_6_5
QSK_6_5
KX_6_5
KT_6_5
BQ_6_11
QSK_6_11
KX_6_11
KT_6_11
BQ_6_23
QSK_6_23
KX_6_23
KT_6_23
Gammaproteobacteria
Actinobacteria
Bacteroidia
Cyanobacteriia
Alphaproteobacteria
Acidimicrobiia
Clostridia
Planctomycetes
Verrucomicrobiia
norank_Bacteria
Bacilli
Armatimonadia
Deinococci
norank_p__SAR324
Oligoflexia
Thermoleophilia
SL56_marine_group
Phycisphaerae
Polyangiia
Vicinamibacteria
Chlamydiia
unclassified_p__Pseudomonadota
Fimbriimonadia
Myxococcia
Bacteriovoracia
Babeliae
Kapabacteria
OM190
Acidobacteriae
Gemmatimonadia
others
